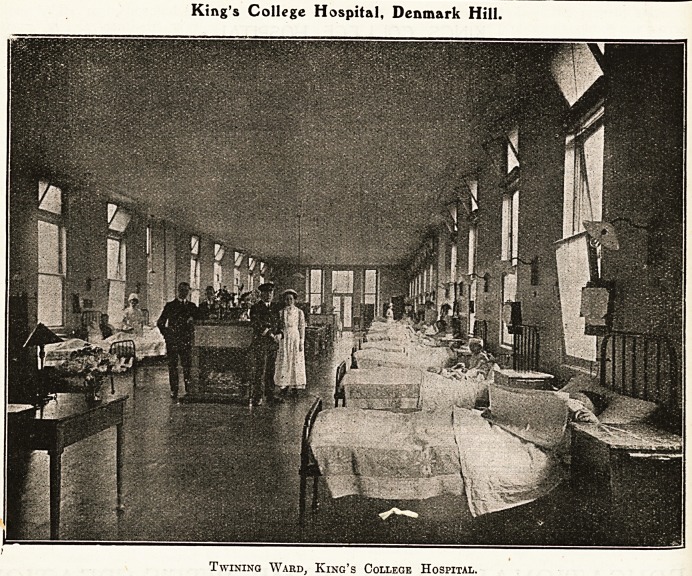# The New King's College Hospital, London

**Published:** 1915-12-18

**Authors:** 


MAIN ENTRANCE FROM BESSEMER ROAD.

THE ADMINISTRATION BLOCK.

### THE NEW KING'S COLLEGE HOSPITAL, LONDON.

Having dealt with a few vital factors which have centred round the re-creation of several of the older London hospitals, we will now proceed to consider a new departure arising from the demand of the public to re-arrange the distribution of several large general hospitals in London by transferring them from old central sites to new ones situated on the outskirts—that is, in the Greater London area—so as to meet the changed conditions of modern methods of quick and cheap transit from and to all parts of the Metropolis, and provide that each largely populated district may have a great general hospital situated near its centre. To accede to this demand meant a revolution in procedure, for it necessitated the setting aside of old traditions and the facing of great financial problems involving the raising and expenditure of enormous sums upon new buildings. The King's Hospital Fund has from the outset shown favour to this movement for partial decentralisation, and when it was found impossible to continue successfully the work of King's College Hospital on its old site in the neighbourhood of the Law Courts the Council warmly supported a proposal to rebuild this hospital on a new site, which was given a tremendous impetus by the generosity of Lord Hambleden (then the Hon. W. F. D. Smith), who, in 1904, when chairman of the King's College Hospital Board of Management and the Removal Fund Committee, acquired and presented a site of just over twelve acres in South-East London. This land was formerly occupied by six large comfortable houses, with delightful gardens, orchards, and meadows. It is rectangular in shape, has a gentle slope from east to west, and a marked fall from south to north of some 15 feet. It is situated at the junction of Denmark Hill with the South-Eastern and Chatham and the London, Brighton and South Coast Railways, whilst Ruskin Park of 22 acres stands immediately opposite to it on the other side of the railway. The site occupies a high and healthy situation, and is in proximity to a fair amount of open country, or, rather, of ground occupied but thinly by roomy houses with large gardens and fields. The proportions of unoccupied land to buildings is, land 7.75 acres, buildings 4.50 acres. On the completion of the hospital buildings it was divided into two sections by the main hospital corridor—*i.e.*, into a northern and a southern portion. The ward pavilions occupy the whole of the southern portion of the site, stretching from east to west in an unbroken line, the chapel and central station bisecting this, as will be seen on reference to the block plan, page 247. Every ward has consequently the benefit of an abundance of sun and air, whilst the outlook from the wards over Ruskin Park is a marked advantage and has attractions.

Energetic efforts were made to raise the large sum of £300,000, which was required, in addition to other funds in hand, to defray the cost of providing a new, up-to-date hospital at Denmark Hill. Towards this sum, at the end of 1914, the King's Hospital Fund had contributed £56,000. How much of the balance has been obtained from the public cannot be stated, for the last report of the hospital to December 31, 1914, contains no information on the point. The expenditure on the land, buildings, and equipment of the new hospital is entered in the balance sheet in that report as upwards of £552,000. The number of beds provided for is 358, of which 152 are at present open for the accommodation of civilian patients. Under an arrangement between the hospital and the War Office providing for the admission of wounded warriors, it has been found possible to accommodate in the new buildings upwards of 700 patients, together with the increased staff which these numbers entail. In passing, we may remark that the absence from the report of King's College Hospital of any clear statement and precise information of the position of the Building Fund accounts and of the amount of money still required to liquidate the whole of the outstanding liabilities cannot fail to affect adversely the prompt provision of the necessary funds by the public.

On March 17, 1908, Lady Esther Smith cut the first sod after a brief dedicatory service, and building operations were commenced. On July 20, 1909, King Edward VII. and Queen Alexandra attended in semi-state, and His Majesty laid the foundation stone, which occupies a position in the main entrance portico. This action of King Edward VII., as our narrative testifies, is of special historical value, which is deepened in interest by the fact that it was the last public ceremony of the kind which His Majesty attended before his lamented death. On July 26, 1913, King George V. and Her Majesty the Queen inaugurated the new buildings, to which patients were first admitted in the following November.

The occupation by wounded soldiers of the larger portion of this great hospital, owing to the war, has necessarily set back and delayed the completion of its organisation as a civil hospital and medical school. This circumstance, coupled with the fact that Mr. William A. Pite, the architect, has published in the *Journal* of the Royal Institute of British Architects, April 3, 1915, an exhaustive and descriptive account of the new hospital buildings, their construction and equipment, with plans and illustrations, has decided us not to attempt any further detailed description at the present stage of the organisation. We propose to confine our narrative to a consideration of the extent to which the new King's College Hospital constitutes a departure in conception and planning.

### Hospital Planning at its Best.

Mr. Pite possesses the invaluable gift of taking infinite pains. When he was entrusted with the commission to provide London with the latest, most up-to-date and complete hospital buildings possible, including those for a medical school, a special pathological block, an unusually spacious out-patient department, a bathing establishment, and extensive casualties accommodation, he evidently made up his mind to set himself to the creation of a type of hospital which would mark a new departure and take a first place when compared with existing British hospitals. This policy of the architect's has been pursued with untiring zeal, and has entailed upon him an enormous amount of extra labour and special thought and study. Mr. Pite has evidently sought the counsel of those who had an intimate personal knowledge from actual and recent experience of the ever-increasing requirements which new conditions and innumerable developments in modern science have caused in methods of treatment. This is abundantly evidenced in the departments and units of this hospital. The result as a whole justifies the brains and labour expended upon the work, for the new King's College Hospital is undoubtedly a creation which everybody who wishes to learn how to cover the maximum of ground demanded by modern conditions in hospital construction should study and master. The buildings present many new features in detail, and it is satisfactory to find that tradition has not been allowed, so far as we could discover, to interfere with or prevent a full carrying out of the whole conception of what new hospital buildings in these days should include. All honour to Mr. Pite, and warm and hearty congratulations and thanks to him for the service he has thus rendered in making a distinct advance towards the ideals which must ever be the aim of the most capable administrators and architects in collaboration, and of most practical and efficient members of the profession of medicine too.

For the present, under existing conditions, King's College Hospital, as we have already stated, cannot be administered on the highest lines of a great civilian hospital, nor will this be possible until the termination of the war and the departure of the warrior patients. It follows that it may be well for those who wish to study this hospital in full working order and on its merits to delay their inspection until the present access of patients and mixed conditions of management have terminated.

### Not Critical, but Descriptive.

We now come to the consideration of some of the notable features which the new King's College Hospital presents. It must be understood that we are not, for the reasons indicated, in a position to attempt a criticism, and that our object is to give

(*Continued on page* 248.)

**King's College Hospital, Denmark Hill.**

Twining Ward, King's College Hospital.

### HOSPITAL PLANNING AT ITS BEST *(continued from p. 246).*

a terse review of the latest attempt to produce a great modern hospital, full of new and suggestive features.

The general plan of the main ward pavilions, of which there are eight, has already been indicated, and to the description may be added the fact that the special ward pavilions are placed on the site to the north-west at the extreme end of the main corridor. The administration block is situated in the centre of the northern portion of the site, facing Bessemer Road. It consists of four portions, the front one containing a vestibule, on either side of which are the porters' office and students' room. Beyond this is a transverse corridor, the portion to the left leading to the nursing home, and that to the right to the resident medical officers' quarters, the separate and private entrance to which is at the north-west corner of the administration block. The former is shut off by doors placed across the corridor, which is always kept cut off by a locked door. Beyond the corridor opposite the lobby is the hall, out of which runs a corridor to the principal staircase, which the architect describes as "the eye or centre" of his whole scheme. On either side of this corridor are placed the secretarial department and the board-room accommodation. The resident medical officers are well-housed in the west wing, where bed and sitting room accommodation is provided for twelve residents, every two of whom have the use of a common sitting-room. Three bath-rooms and a sanitary annexe are set aside for the use of the resident medical staff. There are in addition medical staff dining and recreation rooms which are placed on the ground floor in front of the building looking out on to Bessemer Road. On the left of the vestibule, on entering the corridor, as already mentioned, is placed the nurses' home, the private entrance to which is at the north-east corner of the administration block, where there are a female porter's office, a cloak-room and lavatory, and a room in which nurses can see their friends. Here, too, is installed an electric push-button lift communicating with all the floors. On the ground floor is also provided the large nursing staff dining-hall, 63 feet by 38½ feet, with seating accommodation for 150 nurses, together with a serving-room, hot plate and carving table, also electric lifts communicating with the kitchen department below. This dining-hall is attractive and in every way good, whilst the adjacent provision of two small mess-rooms, one for the use of sisters and the other as a luncheon-room for the nurses, is commendable on every ground.

## Figures and Tables

**Figure f1:**
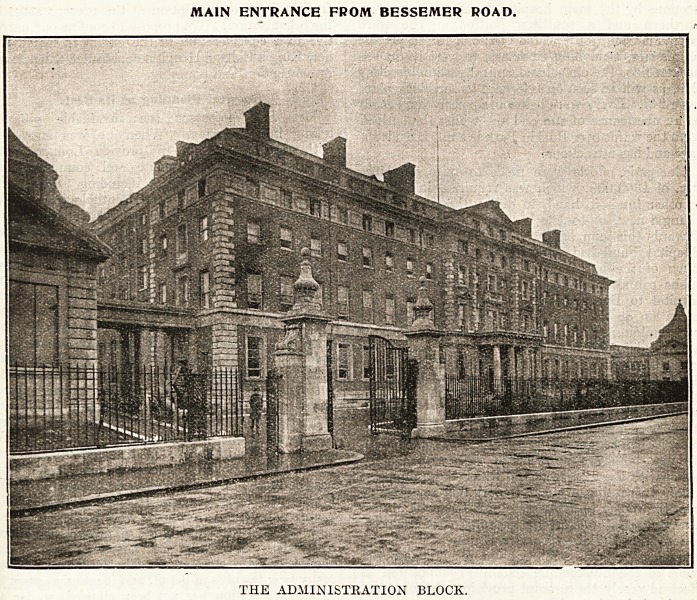


**Figure f2:**